# Vaccination in the Light of the Royal British Commission

**Published:** 1897-06

**Authors:** M. R. Leverson

**Affiliations:** Fort Hamilton, L. I., N. Y.


### VACCINATION IN THE LIGHT OF THE ROYAL BRITISH COMMISSION.

M. R. LEVERSON, M. D., FORT HAMILTON, L. I., N. Y.

(Continued from page 160.)

The next witness was Staff Surgeon Theodore J. Preston, M. D., of the Royal Navy. He testified that vaccination was made compulsory in 1864, but that this order did not extend to Kroomen until 1873. He furnished a table (Table B, App. 5, Second Rep. of the R. C., p. 254) showing the number of attacks and of deaths from small-pox in the Royal Navy for each of the years 1860-88, with, in each case, the ratio per 1,000 of force. From this table the following table has been extracted and compiled:

| Years. | Mean Force. | Small-pox Attacks. No. | Small-pox Attacks. Ratio per 1,000 of Force. | Deaths. | Fatality per cent. of Cases. |
|---|---|---|---|---|---|
| 1865 | 51,210 | 164 | 3.2 | 15 | 9 |
| 1866 | 49,475 | 239 | 4.9 | 8 | 3.5 |
| 1867 | 51,000 | 251 | 4.9 | 14 | 5 5 |
| 1868 | 51,220 | 83 | 1.6 | 2 | 2.5 |
| 1869 | 48,820 | 82 | 1.6 | 5 | 6.1 |
| 1870 | 46,710 | 40 | .8 | 1 | 2.5 |
| 1871 | 47,460 | 148 | 3.1 | 12 | 8 |
| 1872 | 46,830 | 84 | 1.9 | 11 | 12.4 |
| 1873 | 45,440 | 16 | .3 | 1 | 6 |
| 1874 | 44,530 | 9 | .2 | 1 | 11 |
| 1875 | 44,360 | 17 | .3 | 1 | 6 |
| 1876 | 45,010 | 24 | .53 | 6 | 25 |
| 1877 | 44,940 | 17 | .37 | ... | ... |
| 1878 | 46,400 | 9 | .19 | ... | ... |
| 1879 | 44,745 | 52 | 1.11 | 14 | 27 |
| 1880 | 44,770 | 7 | .15 | 1 | 15 |
| 1881 | 44,400 | 25 | .56 | 3 | 12 |
| 1882 | 43,475 | 10 | .23 | 2 | 20 |
| 1883 | 43,350 | 9 | .27 | ... | ... |
| 1884 | 43,000 | 6 | .13 | ... | ... |
| 1885 | 46,670 | 7 | .14 | ... | ... |
| 1886 | 46,770 | 11 | .23 | 3 | 27 |
| 1887 | 48,410 | 1 | .02 | ... | ... |
| 1888 | 50,060 | 19 | .36 | 1 | 5 |

Is it possible for any one to fail to observe from this table the total indifference of both attacks and fatality to vaccina tion! It was attempted to be shown (Q. 2,659 and 2,668) that most of the cases occurred among the Kroomen of the west coast of Africa and the natives of India, whose vaccination was not compelled until 1873. But in 1879 there were fifty-two cases, thirty-nine on the west coast of Africa, and Dr. Preston said (2,668) "these would be nearly all Kroomen," and (2,699) in reply to Professor Michael Foster, "All probably unvaccinated at that date; they are compelled to be vaccinated now." Thereupon Dr. Collins asked him the date of the order compelling foreigners who joined the navy abroad to be vaccinated or re-vaccinated, to which Dr. Preston answered 1873. But the fifty-two cases as to which Dr. Preston answered as above occurred in 1879!

The foregoing took place at the session of the Commission on October 30th, 1888, and at the session held on the 8th of November the subject was resumed. The "Boadicea," the flagship of the west coast of Africa, had thirty-nine cases of small-pox on board in 1879 (3,153). She had thirty-seven Kroomen as part of her crew, of whom sixteen had small-pox, and all the rest had been vaccinated, but, says Dr. Preston, they had not been re-vaccinated. With regard to the white men Dr. Preston says they "had the disease in a modified form." But (Q. 3,155) "one white man died." Was his death also modified?

Upon being questioned by Professor Michael Foster (3,157), "But so late as 1879 the Kroomen were vaccinated, too, were they not?" instead of answering the question in a straightforward manner as his answer to (2,699) required, he now says, "They should have been re-vaccinated."

Then, instead of the fact being as stated by Dr. Preston (2,668), "these would be nearly all Kroomen," it now turns out that only nine were Kroomen (3,177). Upon being questioned by Mr. Picton (3,283) upon the "Report of the Health of the Navy for 1881," it appeared that the first case on board the "Eclipse" was that of a leading seaman (white), aged thirty-one, who had been re-vaccinated two years previously. His was a severe case of the confluent form, and he died on the ninth day (28th of April). On the 29th of April a second case occurred, also a white man, aged twenty-seven, successfully re-vaccinated four years previously; also a confluent case, and he died on the eleventh day of the disease. So that the first two cases at least on board the "Eclipse" that occurred were both re-vaccinated cases, and they died. It is difficult to see how either of these men had been "protected" or had their attacks "mitigated," or their deaths "modified" by either vaccination or re-vaccination.

Surgeon Preston suggests that probably the re-vaccination of one of them had been "imperfectly performed." An observation which recalls Jenner's "spurious cow-pox," which Cobbett was cruel enough to define as one of the shuffles of quackery. Four cases occurred on the China Station in 1883; all had been vaccinated, and three of them "successfully re-vaccinated." In the case of the fourth the vaccination "did not take." (3,270.) But according to "the authorities," the "not taking" is evidence of full protection, and one of these authorities has invented the phrase "Vaccinization," to denote vaccinated until vaccination will no longer take, and only then is protection perfect!

At (Q. 3,331) Dr. Collins took up the case of the Kroomen (Sup. 2,668). Dr. Preston now repeats what he had just said, that instead of the thirty-nine cases being nearly all of them Kroomen, only nine were, and explains that "evidently he did not understand the question as it was put." Without reproducing the entire examination it would be impossible to do justice to this excuse; this I do not think it worth while to do. Without assuming that Dr. Preston desired to state what was untrue, it is evident that he was under so strong a bias that he snapped at what seemed to him "a way out," and not only assumed that the patients were Kroomen, but also that they were unvaccinated, both of which statements he took back on the 8th of November, and at (3,333) and (3,334) he admits that twenty-eight of the cases were white men, and he states (3,335) "the one white man who died with confluent small-pox had two infantile vaccination cicatrices only." (3,336.) "Were they well marked? Presumably not, but I have no documentary evidence upon that point." Thereupon at (3,338) the "Navy Report of the West Coast of Africa Station, 1879," is produced, showing that "of the twenty-five cases received, one white man, who had two well-marked vaccinal cicatrices on his arm died of the hemorrhagic form of the confluent variety." His vaccination had evidently "mitigated" the small-pox in his case! As he died twelve years after the rigorous enforcement of vaccination and re-vaccination in the British Navy, the statement (3,335) that he had only two infantile vaccination cicatrices cannot correctly represent the facts. The commander of the "Boadicea" (a much vaccinated white man) had small-pox of the confluent variety, as did also three other white men of the crew. (3,340-1-2.) Mr. Meadows White takes up the examination at (3,345). Dr. Preston had stated (Q. 3,154-5) that out of twelve deaths from small-pox on the west coast of Africa in 1879 eleven were deaths of Kroomen, but it now turns out that seven were Kroomen and five white men (3,347); and (3,348) Dr. Collins elicits the fact that all the Kroomen who died had had small-pox previously except the two who had vaccine marks on their arms, besides "doubtful scars on different parts of their bodies." (3,350.)

A curious piece of pathology was testified to by Staff Surgeon Preston. He stated, in answer to Sir James Paget (3,361), that "about twenty years ago in some of the cases of small-pox that took place, I think, on the South American Station, that an eruption of syphilis appeared to have some modifying effect upon the eruption of small-pox; but that was in the pre-vaccination days."*

* Under the head of "remarks" in Table B before referred to, we are informed under the date 1860, " Vaccination recommended by the Medical Director General," and under the date 1864, "Admiralty orders all new entries to be vaccinated;" yet Dr. Preston now refers to twenty years ago—*i. e.*, to 1869 as "pre-vaccination days."

(Q. 3,362.) "Nothing of the kind, so far as you know, has been brought out by re-vaccination?" "Nothing of the kind so far as I know."

It is an undoubted pathological law that the presence of a constitutional disease will prevent the occurrence in the same patient of another *similar* constitutional disease, and will modify a different constitutional disease. If, as not merely admitted, but boastingly asserted by the champions of vaccination, Vaccinia is a constitutional disease, it must prevent the occurrence of a similar and modify a dissimilar constitutional disease. It is confessed now by the champions of vaccination that vaccination does not always prevent, but they claim that where it does not prevent it at least modifies small-pox. Dr. Preston himself is of this number, for while arguing before the Commission in favor of re-vaccination he said (Q. 3,386), "These 5,000 odd men who presented a perfect vaccine vesicle I should say were susceptible to small-pox, but probably in a modified degree."

If now this contention be true it presents a strong argument in favor of the views of those who, having studied the pathology of cow-pox, declare its human analogue to be syphilis, and not small-pox, for, says Dr. Preston, the presence of syphilis was observed by medical officers in pre-vaccination days to modify small-pox. Yet, as before quoted (Q. 3,362), he knows nothing of the kind (*i. e.*, a modification of small-pox) to have been brought about by re-vaccination. Does it not seem as though when it becomes necessary "to preserve vaccination from reproach" when it has wholly failed to protect, he finds that it has modified the disease; but when this brings close to view the true analogue of vaccinia in syphilis he has never seen it?

A curious insight into the utter and irreconcilable differences of opinion among the various champions of vaccination respecting (1) the sort of poison to be employed in vaccinating, (2) the mode of operation, (3) the number of marks, (4) the duration of the protection, etc., etc., is to be obtained by a careful perusal of these reports. It is impossible to give a correct idea of it in a mere abstract.

Our readers will remember Sir John Simon's opinion, coinciding with Jenner's, that the protection was life-long, or nearly so, and *that* is the only orthodox belief upon the subject. Now comes Dr. Preston and objects to this; so does (Q. 3,390) Dr. Macdonald, Medical Officer on board the "Lord Warden," who claims that primary vaccination is exhausted in seven years, and that successful re-vaccination after seven years implies an almost complete absence of protection and mitigation (Q. 3,393); but to this Dr. Preston objects (Q. 3,392). Dr. Seaton differed entirely from Dr. Macdonald, and advocated "re-vaccination about or after puberty where the primary vaccination has been anything short of Marson's highest class (Seaton's *Hand-Book of Vaccination*, p. 303); but it seems also certain that a re-vaccination at or after this period of life may give additional security to many whose original vaccination has been complete." Sir Wm. Jenner concurs with Dr. Preston, but also wishes re-vaccination whenever there is an epidemic, to which Dr. Guy demurs; Dr. Oakes wants it repeated every ten years; Dr. Thorpe Porter declares re-vaccination is all nonsense, and Dr. Pringle says that re-vaccination every seven years, or whenever there is a small-pox scare, is unphysiological and unpathological.

The next witness was Dr. Thomas W. Grimshaw, Registrar-General of Ireland. He states (Q. 2,707) that vaccination was first made compulsory in 1864, and handed in a table (App. VI, Table A, p. 256, of Second Report R. B. C.) showing for all Ireland the estimated population in each of the years 1864-88, the number of deaths from small-pox in each year, and the rate per million living. He very properly included cases of alleged chicken-pox because he considered that they were most probably cases of small-pox (2,721), and here another curious fact must be mentioned. It is universally considered by all physicians that chicken-pox never kills. It was not until physicians found that vaccinated persons did really die of small-pox—notwithstanding that Dr. Jenner observed that no vaccinated person "could by any possibility catch the small-pox," or as later revised by him and Simon, "could by any possibility die of it," that deaths from undoubted small-pox were certified to as "chicken-pox" "to save vaccination from reproach."

Dr. Grimshaw states (2,730-1-2): In 1858 an act was passed to make further provision for the practice of vaccination in Ireland. In 1864 vaccination was made compulsory.

The following table, taken in part from Table A, above mentioned, and in part from Table C (App. No. 6, Second Report R. B. C., p. 257), gives the deaths from small-pox per million living for each year from 1864 to 1888, inclusive:

| Years. | Estimated Population. | Births. | Persons Vaccinated. | Deaths per 1,000,000 Living. (Table A.) |
|---|---|---|---|---|
| (From Table A.) | | (From Table C.) | | |
| 1864 | 5,640,527 | 136,414 | 191,810 | 151.4 |
| 1865 | 5,594.589 | 144,970 | 169,142 | 82.4 |
| 1866 | 5 522,942 | 146,090 | 137,124 | 35.1 |
| 1867 | 5,486,509 | 144,388 | 125,741 | 3.8 |
| 1868 | 5,465,914 | 146,051 | 131,426 | 4.2 |
| 1869 | 5,449,094 | 145,659 | 125,672 | 3.7 |
| 1870 | 5,418.512 | 149.846 | 140,220 | 5.9 |
| 1871 | 5,398,179 | 151,355 | 179,889 | 123.2 |
| 1872 | 5,372,890 | 149,278 | 282,484 | 604.5 |
| 1873 | 5,327,938 | 144,377 | 138,873 | 94.6 |
| 1874 | 5,298,979 | 141.288 | 139,587 | 107.3 |
| 1875 | 5,278,629 | 138,320 | 137,340 | 101.4 |
| 1876 | 5,277,544 | 140,469 | 114,487 | 4.5 |
| 1877 | 5,286,380 | 139,659 | 116,679 | 13 4 |
| 1878 | 5,282,246 | 134,117 | 133,045 | 165.3 |
| 1879 | 5,265,625 | 135,328 | 126,911 | 127.6 |
| 1880 | 5,202,648 | 128,086 | 147,828 | 74.8 |
| 1881 | 5,144,983 | 125,847 | 113,557 | 15 7 |
| 1882 | 5,097,853 | 122,648 | 132,825 | 26.9 |
| 1883 | 5,015,282 | 118,163 | 106,071 | 5.8 |
| 1884 | 4,962,693 | 118,875 | 102,548 | 0.8 |
| 1885 | 4,924,342 | 115,951 | 102,312 | 2. |
| 1886 | 4.889.498 | 113,927 | 94.861 | 1. |
| 1887 | 4,837,313 | 112,400 | 96,489 | 4.1 |
| 1888 | 4,777,534 | 109,557 | 92,498 | 1.7 |

The above table shows to all but those who will not see how regardless of vaccination was the mortality from small-pox in each of the years cited by Dr. Grimshaw. But a study of the diagram put in by Dr. Grimshaw in connection with his Table C brings out another fact,* viz., that the deaths from small-pox increase as vaccination increases, and as the number of vaccinations diminish so, too, do the deaths from small-pox. The variations from this rule are slight and irregular.

But the facts become still more noteworthy when we read this diagram in connection with Dr. Grimshaw's answer (3,028), "The births and deaths years are made up to the 31st of December; the local government year ends on the 30th of September, and it is on the latter that the record of public vaccinations is published."

Thus the deaths recorded as occurring in any year are the deaths which occurred from the 1st of January to the 31st of December of that year, inclusive, while the vaccinations recorded as belonging to that same year are the vaccinations which were performed from the 1st of October to the 30th of September previously. The records, then, of small-pox deaths, as shown from the testimony of an ardent advocate of vaccination, from the records kept and statistics compiled by himself, and as shown in the diagram prepared by himself, establish not merely a synchronism of lower small-pox deaths with less vaccination, and of higher small-pox deaths with more vaccination, but that the lower small-pox deaths are sequences to less vaccination, and that the higher small-pox deaths are sequences to more vaccination!

At (2,863) Dr. Grimshaw furnishes a report of hospital small-pox cases personally observed by him. From 1871

---

* It is very desirable that this and other diagrams should be published in connection with the abstract now publishing in THE HOMEOPATHIC PHYSICIAN. Whether this can be done or not will depend upon the responses made to the invitation which will be found on p. 226 of this number of THE HOMEOPATHIC PHYSICIAN.

to 1886 there were 3,860 cases in the Cork Street Hospital. There were 2,217 discrete cases, 1,245 were confluent and 398 malignant cases. Of the 2,217 discrete cases 94.9 per cent. were vaccinated; of the 1,245 confluent cases 64.4 per cent. were vaccinated; of the 398 malignant cases 243, or 61.1 per cent., were vaccinated; of all the 3,860 cases 822, or 21.3 per cent., died. Of the 822 fatal cases 368, or 44.8 per cent., were vaccinated, and 454, or 55.2 per cent. unvaccinated. Can it be said that vaccination either prevented small-pox or mitigated it in the case of the admittedly vaccinated 368 who died?

Dr. Grimshaw, in connection with his answer (2,863), handed in a table (Table G, p. 272, Second Rep. R. B. C.), showing some statistics of small-pox cases in the Cork Street Hospital. In this he gives the total number of vaccinated malignant cases at 243, with deaths 168, or 69.1 per cent. of fatality.

Dr. Grimshaw deals in the same legendary figures with regard to the fatality of the unvaccinated, as preceding witnesses had done; but as he gets a total fatality of 21.3 per cent. of deaths on the aggregate of cases, and the hospital mortality in pre-vaccination days was only 18.8 per cent., his assertion (Q. 2,683, p. 87a) that the fatality of the unvaccinated reached 64 per cent. is clearly absurd.

Neither he nor any other of the advocates of vaccination has ever attempted to grapple with the problem: "Since the pre-vaccination fatality in hospital cases was 18.8 per cent., and 64 minus 18.8 equals 45.2, who kill this 45.2 per cent.?" A large number of so-called unvaccinated deaths consisted doubtless of children under the vaccination age, small-pox in children under three (or even six) months being nearly always fatal under the treatment of allopathic physicians; another large number is in most cases to be accounted for in the fact that according to the statements of nearly all the compilers of these statistics they only recorded those cases as vaccinated on whom the marks were visible; but as most of the severe cases are confluent cases, in whom the marks are generally obscured and invisible, these severe cases would go down among the unvaccinated unless they recovered, when the marks becoming visible, they would be transferred to the vaccinated list, and "saved by vaccination." But Dr. Grimshaw states that he was guided by the statements of the patient or his friends, and there must be some other explanation of Dr. Grimshaw's figures. It is unfortunate that with all the particulars detailed by Dr. Grimshaw he omitted to give the condition as to vaccination of cases and deaths, age for age, as was done by Dr. Keller, head physician of the Austrian State Railway Company, for the years 1872-3.

Dr. Grimshaw further shows (2,866) by Table J (App. 6, p. 274, Second Rep. R. B. C.) that whereas the average deaths from small-pox of children under five years of age during the period 1864-68 was at the rate of 52.7 per cent. of the small-pox deaths, in the subsequent quinquennia it was only from 24.9 to 25.8 per cent.*

This is in line with the hypothesis before commented on, that small-pox is now more a disease of mature age than it was formerly; but it cannot be said to be valid evidence thereof until some other factors of the problem can be determined, some of which must for the present, at any rate, be given up as insoluble.

In the first place, we should need to know what was formerly the small-pox death-rate of children under five years of age, and the proportion of that death-rate to the small-pox mortality at all ages, also the total death-rate of such children, and its ratio to the all-age mortality.

If the total death-rate of such children is not reduced in at least as great proportion as the reduction in their deaths from small-pox, then whatever saving occurred in children's

---

* See on this subject *supra*, p. 115-16, and *post*, p. 224, Q. 3,004.

deaths from small-pox must have been made up by other causes, and it becomes probable that such causes were vaccination, and the diseases induced by vaccination. This probability will be much increased should it appear that deaths from inoculable diseases among children have largely increased. If the child death-rate and the small-pox child death-rate have both diminished there will then be the further question, How much of this reduction is due to better sanitation, better nourishment, better clothing, etc.? Further, if other diseases have diminished in greater, or even in an equal degree with small-pox, why should vaccination be claimed as producing the saving in small-pox rather than in such other diseases?

Dr. Grimshaw put in a table (Q. 2,864) (Table H, App. VI, Second Rep. R. B. C., p. 273) showing the deaths in Dublin from all causes, and (Q. 2,865) he thinks "There can be little doubt that where small-pox prevails in the epidemic form it materially increases the general death-rate." To illustrate this he put in a diagram (opposite p. 274, Second Rep. R. B. C.)* An inspection of this diagram seems to bear out Dr. Grimshaw's conclusion only in the case of the year 1872. In 1866, 1883, 1885, and 1887 the total mortality was very high, and the small-pox mortality very low. In 1878 the small-pox mortality was high, and, although the total mortality was above the average, it can hardly be called high. This diagram also shows in a very striking manner the small share in the total mortality borne by small-pox, even in epidemic years, and the very great one borne by diseases of the respiratory organs and consumption.

In answer to (Q. 2,868) Dr. Grimshaw puts in a table of seven deaths from cow-pox and other effects of vaccination from 1st of January, 1881, to 30th of September, 1889. Let us understand what this admission means. It means that

---

*See note to p. 213 with reference to the reproduction of this diagram.

seven persons have been deliberately done to death with exquisite tortures who would otherwise have lived in the ordinary enjoyment of life and health. They are not put to death for any offense against society, nor to save others from death or injury (though the killing of one of a starving crew to keep the rest alive is denounced as a crime, both by morals and by law). These seven admitted killings under torture (forming but a small fraction of those which in truth occur) were perpetrated under the pretense of thereby preserving the victims themselves from being attacked by a disease they might never get, and which if they should get is, as Sydenham justly said over 200 years ago, "the slightest and safest of all diseases," provided "no mischief be done by physician or nurse," and which, with the exception only of persons of low vitality, can become fatal only through the "mistakes of physician or nurse."

At (2,869) Dr. Grimshaw thus endeavors to explain away some of these cases: "In Case 3 the certificate is, Vaccinia sixteen days, debility five days. There is nothing to show any connection between the debility and the vaccinia. Case 5 is manifestly a death from enteritis, a common cause of death in infants affected by diarrhea, but there is nothing to show that the vaccination had any connection with the enteritis, especially as it did not arise until twenty-eight days after the vaccination. With regard to Case 6 there is some question whether that was not a case described as cow-pox without its having any relation to vaccination at all.* Case 7 was eczema and boils; the former is a very common disease among infants, and did not arise until a month after the vaccination; therefore it can scarcely have had any connection with it."

---

* In his table of these cases given in (2,868) the case is thus given:

| Name. | Locality. | Date of death. | Stated cause of death | Age at death. |
|---|---|---|---|---|
| 6. Mary Devers. | Ballino, Co. Mayo. | 9–10–'87. | Cow-pock 1 month. | 8 months. |

So that a child of 7 months is vaccinated, gets the cow-pock, and dies at 8 months—yet Dr. Grimshaw *questions* whether it had any relation to vaccination!

Comment upon these attempted explanations cannot be necessary, especially when the reader bears in mind the fact that the positive and emphatic order of the Local Government Board is to vaccinate no child that is not to all appearance perfectly healthy.

With regard to infantile syphilis (Q. 2,870) Dr. Grimshaw put in a table (Table M, App. 6, p. 275, Second Rep. R. B. C.) which shows a fluctuating but general decrease of that cause of death per million living in Ireland during the years 1864-1888. To be of weight to offset the conclusion drawn from the increase of infantile syphilis in Great Britain it should be supplemented by one giving the deaths from syphilis at all ages, as well as the deaths from all causes for each of the years referred to in his statistics. A diminution of the general death-rate exceeding the diminution of death from infantile syphilis, or a decrease in adult syphilis to a greater extent than infantile, would not only show that Table M did not in any wise contradict the conclusions drawn from the increase of infantile syphilis in Great Britain, but would, on the contrary, reinforce them. Unfortunately none of the reports of the Commission yet published furnish these statistics. There is, however, a table furnished by Dr. Grimshaw (Table L, App. 6, p. 274, R. B. C.) which, taken in connection with the population as given in Table A (see above, p. 212), will help us to a conclusion. Table L gives the average annual deaths from typhus, typhoid, and continued fever in quinquennial periods for each quinquennium for the twenty-five years 1864-88. From these can be calculated the death-rates per million living from those fevers, which were as follow:

| Quinquennia. | Mean Population. | Mean Deaths from Fevers per 1,000,000 living. |
|---|---|---|
| 1864–1868, | 5,538,096.2 | 793 |
| 1869–1873, | 5,393,322.6 | 593.8 |
| 1874–1878, | 5,284,755.6 | 549.1 |
| 1879–1883, | 5,145,278.2 | 501.8 |
| 1884–1888, | 4,818,276 | 334.7 |

Now the average annual deaths from syphilis of children under five years of age per million living, calculated from Table M (App. No. 6, p. 275, *ubi sup.*) is as follows:

| Quinquennia. | Average Annual Deaths from Syphilis of Children under Five Years of Age per 1,000,000 living (Ireland). |
|---|---|
| 1864–1868, | 145.58 |
| 1869–1873, | 97.66 |
| 1874–1878, | 82.36 |
| 1879–1883, | 97.92 |
| 1884–1888, | 84.42 |

Showing that while the ratio of deaths from fevers per million living has fallen during the twenty-five years more than fifty-eight per cent., that of infantile syphilis has only fallen forty-two per cent.

(Q. 2,872.) The proportion of those who became blind from small-pox to the total number of blind fell from 1 in 9.5 in 1861 to 1 in 17 in 1881. Assuming these figures to be correct, and the years to be fairly representative, the rational conclusion must be that physicians have learned something in their treatment of small-pox between 1861 and 1881. Dr. Grimshaw himself gives at (3,019) what seems a sufficient explanation, as he says, "Ophthalmia, which was very prevalent in Ireland, has decreased. . . . If a person suffering from ophthalmia is attacked by small-pox, the ophthalmia attacks the eyes with special virulence." As a matter of fact, blindness only follows small-pox from an omission to observe and treat a pustule on the cornea. Homeopaths know well how to treat it; and even the treatment prescribed by the old school is generally successful to avert blindness. Mr. Pickering's treatment is also always successful.

We are told (Q. 2,881) that "inoculation was practiced to a considerable extent up to the year 1874-75, but on Mr. Whithead asking (Q. 2,884), "If a considerable number of unvaccinated children were inoculated it would go far to destroy the value of any comparison as to the amount of security given by vaccination, would it not?" he answers, "If there were a very considerable proportion of such cases, but I do not think there was any large proportion."

He had made a report for the *Sanitary Record*, in 1875, touching a severe epidemic of small-pox in Athenry in that year (Q. 2,838), and his attention is called (Q. 2,886) to the conclusions at which he had arrived in that report as follows: "No. 1, That the prevalence of small-pox now existing in Athenry is a portion of an epidemic spread over the north and west of Ireland. No. 2, That the spread of the disease in the west of Ireland has been much accelerated by inoculation." He does not deny these "conclusions," but seeks to explain them away, and to claim that inoculation was not very largely practiced (Q. 2,886-8).

Dr. Grimshaw was unable to explain the falling off in vaccinations in Dublin during the last few years, as shown by his Table D. At (Q. 2,897) Mr. Meadows White said to him, "It looks as if the number of deaths from small-pox was declining, and the proportion of vaccination to births decreasing." To which he answers, "I am afraid we are just ready for another epidemic." At (Q. 2,909) Mr. Picton asks him if he considers his Table C offers "reasonable proof" of the effect of vaccination in diminishing deaths from small-pox? Ans.: "It appears to me that there is." And (2,910), or "When vaccinations fell low we immediately had an epidemic of small-pox—the following year, or the year after." The diagram referred to on p. 213 would bring clearly before the eye of the reader that the exact opposite of Dr. Grimshaw's opinion is the truth. Its study will enable the student to appreciate the value of an opinion formed by a mind so constituted as to imagine that Dr. Grimshaw's Table C furnishes any evidence whatever that "vaccination diminishes deaths from small-pox."

Mr. Picton then (Q. 2,913 to 2,929) called his attention to the year 1870, and he is questioned upon the condition of the population as to vaccination. After admitting certain deductions should be made for children dying under three months, calculated in England at 9 per cent., but taken only at 5 per cent. for Ireland, and for those who are insusceptible, as to whom he can give no estimate, he is asked (Q. 2,929), "That would reduce the number to 142,345 (births), and after making other deductions for children insusceptible I must ask you again whether 135,057 is not a very respectable number of vaccinations?" Ans.: "The figures speak for themselves." At (Q. 2,924) he had stated of 1870, "It was a medium vaccination year, I suppose, scarcely either good or bad." It is not possible in an abstract, which is all we can give of this evidence, to give a realizing view of the shuffling and evasion which the questions and answers picture from Q. 2,913 to Q. 2,929; fully to appreciate them the reader should go to the report itself.

Mr. Picton continued (Q. 2,933), "In 1871 the number of births was 151,355, was it not?" Ans.: "Yes." (Q. 2,934.) "The number of vaccinations of children born since 1864 was 139,053, was it not?" Ans.: "Yes, but that was the number of children vaccinated up to the 30th of the previous September"—*i. e.*, from the 30th of September, 1870, to the 30th of September, 1871.

(Q. 2,935.) "But there was a larger number than in the previous year?" "Yes."

(Q. 2,936.) "And in that year did the number of deaths from small-pox spring from 32 to 665?" "It did."

(Q. 2,937.) "In the next year, 1872, the number of births was 149,278?" "Yes." This is from the 1st of January to the 31st of December, 1872.

(Q. 2,938.) "And the number of vaccinations 142,622?" "Yes." This was from the 30th of September, 1871, to the 30th of September, 1872.

(Q. 2,939.) "And the number of small-pox deaths suddenly rose to 3,248?" "Yes." From 1st of January to 31st of December, 1872.

(Q. 2,942.) "In the following year did the number of vaccinations fall to 119,319 of children born since 1864?" "Yes."

(Q. 2,943.) "Did the number of small-pox deaths fall to 504." "Yes."

(Q. 2944-6.) The amount of vaccination during the next two years was practically the same, and the deaths from small-pox increased in 1874 and fell in 1875.

(Q. 2,947.) In 1876 the number of vaccinations of young people fell to 112,489, and the number of small-pox deaths fell to twenty-four.

(Q. 2,949.) He thinks those figures are consistent with the theory that the increase of vaccination decreases the number of deaths from small-pox because the vaccinations rose because the small-pox was there, not the small-pox because the vaccination was there.

(Q. 2,950.) "That is your explanation?" "Yes, and the converse is very apparent; wherever vaccinations fell very low the small-pox began to show."

(Q. 2,951.) "We will take the years where the number of vaccinations of children born since 1864 has fallen apparently very low; in 1884 it fell to 99,445, did it not?" "Yes."

(Q. 2,952.) "How many small-pox deaths were there then?" "One."

(Q. 2,953.) "Has that low rate of vaccination been since followed by any very great increase of small-pox?" "No: certainly not."

(Q. 2,954.) "In 1888 the number of vaccinations was 89,627?" "Yes."

(Q. 2,955.) "In that year did the number of small-pox deaths largely increase?" "No." There were three. (See Table C, App. 6, p. 257, Second Rep. R. B. C.)

(Q. 2,957.) By Dr. Collins: "Do you attribute the epidemic of 1871 and the following years to neglect of vaccination?" "I do, and re-vaccination."

(Q. 2,958.) "Are you aware that before the Committee of the House of Commons, which sat in 1871, the progressive diminution of the small-pox death-rate in Ireland for 1865 to 1870 was claimed as the result of the operation of the Vaccination Act?" "Yes, I believe it was."

In (Q. 2,959) Dr. Collins quoted to him the statements of the Poor Law Commission of the 27th of September, 1869, read by Dr. Lyon Playfair on that Committee, the opinion of Sir Dominic Corrigan approving thereof, the opinion of Dr. Burke, then Superintendent of Statistics at the General Register Office, to the effect that Ireland was a well-vaccinated country, and that it was owing to such vaccination there was so little small-pox.

(Q. 2,961.) "It was incorrect to represent Ireland as a well-vaccinated country in 1871?" "I think that statement is rather an over-statement; it certainly was not as well vaccinated as it is at present."

(Q. 2,962.) There is no year for which from official figures there appears to have been more deaths from small-pox in Ireland than in 1872, when there were 3,248.

(Q. 2,964.) In 1872 the deaths from small-pox in Dublin were at the rate of 5 per 1,000, a rate exceeding that of any large English town except Sunderland, Norwich, Wolverhampton, and Newcastle-on-Tyne. The deaths in Cork were 1,873, or at the rate of 9.6 per 1,000 of population, which is a higher rate than that of any large town in England.

The rest of the sitting of the 1st of November was given to inquiries as to the continued practice of inoculation in Ireland, already sufficiently noticed.

On the 6th of November, 1889, Dr. Grimshaw was further examined (Q. 2,976). He knew nothing about the nature of the lymph employed in vaccination in Ireland, and referred to Dr. MacCabe, who "will give you any information that is available on that subject."

(Q. 2,181-4.) The Act of 1864 came into operation during the period 1861-70, and small-pox deaths during that period were fifty-two per million living, but during the next decade, 1871-80, the rate rose to 143, and from 1881-88 it has fallen to six.

(Q. 2,985.) He considers the low mortality from small-pox within recent years, together with the fact that vacccination had fallen somewhat, premonitory of a new epidemic of small-pox. He considers that the "6.6 per cent. of births unaccounted for as regards primary vaccinations a considerable proportion, and may afford food for small-pox."

(Q. 2,986-7.) He thinks the operation of the Vaccination Acts, as at present carried out in Ireland, certainly insufficient absolutely to prevent small-pox, and that primary vaccinations will not prevent any community from suffering from small-pox.

(Q. 2,990.) "The general death-rate of Dublin is very high; it is one of the highest in the United Kingdom." (Q. 2,991.) "It has been 35 per 1,000. The yearly average for the period ending 1873 was 26, and it rose for the next decennium to 29.2."

(Q. 2,998.) Table G shows the mortality (fatality) in vaccinated and unvaccinated cases together to be 21.3 per cent. He could not tell how that compares with the unvaccinated fatality in pre-vaccination times. His table gives 63.9 per cent. fatality for the unvaccinated, and 11.7 for the vaccinated, but could not tell anything about the fatality in pre-vaccination times. The absurdity of these figures has been shown above (p. 214) and had been exposed over and over again long before Dr. Grimshaw thus testified. Do these gentlemen purposely keep themselves ignorant of facts which tell against their "opinions"? If not, how would he explain his remaining ignorant of the small-pox fatality in pre-vaccination days, when all were unvaccinated, so as to be unable to compare it with the unvaccinated fatality now, as alleged by the vaccinists?

The legend of the hospital nurses was inquired into (Q. 2,999-3,000), and he is unable to remember the experience recorded in the "medical press" by Mr. Porter with respect to the attendants at the South Dublin sheds in the epidemic of 1872, where the experiment was tried of *not* re-vaccinating the nurses, but stated that in his time everybody in Cork Street Hospital was re-vaccinated except one resident pupil, who refused, and died of small-pox. Neither had he any distinct knowledge of the cases of small-pox among the re-vaccinated students attending the Miseracordia Hospital. (Q. 3,001.) "There was something about it, but I forget the details."

(Q. 3,004) as far as it goes tends to contradict the alleged change in age incidence of small-pox mortality. It relates to a table (Table I, App. 6, p. 274, Second Rep. R. B. C.*) put in by Dr. Grimshaw. The mean small-pox mortality for all ages is shown by that table to have been seventy-two per million living for the period 1864-88, but for the ages 0 to 5 it was 184; from five to ten years it was 80, and from fifteen to twenty-five years it was 93; while from ten to fifteen years and from twenty-five years upwards the rates were less than the mean for all ages.*

(Q. 3,014.) With regard to the opinion given by Sir Dominic Corrigan, as representing Irish medical opinion before the Committee in 1871, that vaccination will not convey syphilis or any poison whatever but itself, Dr. Grimshaw says, "I do not think that that is a universally accepted opinion. I think that many men would hold that other diseases might be conveyed by vaccination;" but (Q. 3,016), "I really have not a very definite opinion upon the subject. I think it is a very doubtful question."

---

* *Supra*, p. 215.

